# Fine Mapping and Candidate Gene Analysis of a Major Locus Controlling Black Seed Coat Color in Mung Bean (*Vigna radiata* L.)

**DOI:** 10.3390/plants15172594

**Published:** 2026-08-25

**Authors:** Dong Deng, Yuning Huang, Yang Zhao, Ming Feng, Jian Chen, Tao Li, Weide Ge, Renfeng Xue

**Affiliations:** Crop Research Institute, Liaoning Academy of Agricultural Sciences, Shenyang 110161, China; dengdong3366@126.com (D.D.); huangyuning0706@163.com (Y.H.); zhaoyang1108@163.com (Y.Z.); fengming718@126.com (M.F.); hellokittycc@126.com (J.C.); wsltao@yeah.net (T.L.); snowweide@163.com (W.G.)

**Keywords:** *Vigna radiata*, seed coat color, *VrScL115*, bulked segregant analysis sequencing, fine mapping

## Abstract

Seed coat color is an important quality trait in mung bean (*Vigna radiata*) and is closely associated with seed appearance, commercial value, and phytochemical composition. To investigate the genetic basis of black seed coat formation, six F_2_ populations were derived from reciprocal crosses between the black-seeded accession LZL115 and the green-seeded accession LZL156. Among 755 F_2_ plants, 559 produced black-coated seeds and 196 produced green-coated seeds, conforming to a 3:1 segregation ratio (*χ*^2^ = 0.37, *p* = 0.54). These results indicated that black seed coat color was dominant and consistent with the control by a single dominant locus, designated *VrScL115*, in the LZL115 × LZL156 genetic background. Bulked segregant analysis sequencing (BSA-seq) initially mapped *VrScL115* to an approximately 2.90 Mb region on chromosome 4. Using newly developed KASP markers and recombinant screening in expanded F_2_ populations, the locus was further delimited to a 121.79 kb interval between markers LS_K3333 and LS_K3379. Of the 11 annotated genes within this interval, *LOC106758748* was the only gene containing high-confidence coding-sequence variants between the parents. This gene encodes a putative R2R3-MYB transcription factor homologous to *MYB90*. Comparative sequence analysis identified several allelic variants potentially associated with black seed coat color, and protein structure prediction indicated local structural differences between the parental proteins. *LOC106758748* showed consistently higher expression in the developing seed coats of LZL115 than in those of LZL156 at 10, 15, and 20 days after pollination, with expression peaking at 15 days. Haplotype analysis of 246 mung bean accessions showed that the LZL115-associated allele combination at LS_K3352, LS_K3365, LS_K3367, and LS_K3370 was present in 21 of 27 black-seeded accessions (77.8%) and absent from all 219 non-black accessions, corresponding to a specificity of 100% and a false-negative rate of 22.2%. These findings support *LOC106758748* as the leading candidate gene for VrScL115; however, direct in vivo functional validation is still required to confirm its causal role in black seed coat formation. The four-marker combination may be useful for identifying germplasm carrying the LZL115-associated allele, although further validation in independent germplasm populations is required.

## 1. Introduction

Seed coat color is an important morphological and quality trait that has been subject to selection during crop domestication and varietal improvement. It directly influences seed appearance, commodity classification, consumer preference, and market acceptance [1,2,3,4]. In mung bean, differences in seed coat color have also been associated with variation in phenolic compounds, flavonoids, pigment composition, and antioxidant activity [5,6,7]. Black-seeded mung bean, in particular, has been reported to possess distinct phytochemical and antioxidant characteristics compared with other seed coat types [5,6,7]. Therefore, elucidating the genetic basis of black seed coat pigmentation is relevant not only to understanding seed appearance traits but also to germplasm characterization and the breeding of mung bean cultivars with specialized market and quality attributes.

Mung bean (*Vigna radiata* L.) is an economically important food legume cultivated extensively in South, East, and Southeast Asia, particularly in India and China [8,9,10]. Considerable variation in seed coat color has been documented in mung bean germplasm, including green, black, yellow, brown, and mottled types [11,12,13]. This phenotypic diversity provides valuable genetic materials for dissecting the genetic basis of seed pigmentation and for selecting germplasm with desirable appearance and quality traits.

Early studies of mung bean seed coat color mainly relied on segregation analysis in populations derived from crosses between parents with contrasting phenotypes. Manguiat et al. [14] reported that green seed coat color was dominant over yellow, whereas Wang et al. [15] found that black was dominant over green and that green was dominant over yellow in the genetic backgrounds examined. These findings indicated that major genes play important roles in controlling contrasting seed coat colors in mung bean. Subsequent development of molecular markers, genetic linkage maps, and increasingly complete mung bean reference genomes has provided the genomic resources required for higher-resolution dissection of seed coat color loci [16,17,18,19,20].

Genome-enabled approaches have identified several genomic regions associated with mung bean seed coat color. Using a diversity panel of 466 cultivated mung bean accessions, Noble et al. [11] identified nine significantly associated SNPs on chromosomes (chr) 3, 4, 5, and 7. A gene encoding an R2R3-MYB transcription factor, *MYB113*, on chr 4 and a gene encoding flavonoid 3′-hydroxylase (*Vrsf3′h1*) on chr 5 were proposed as candidates involved in seed coat pigmentation. Additional loci associated with seed coat color were subsequently reported on chr 2 and 4 [2,21]. Consistent with these genetic findings, pigment analyses detected anthocyanins in black mung bean seed coats but not in green seed coats [22]. More recently, Wang et al. [23] used an F_2_ population derived from a cross between the black-seeded cultivar Jilv9 and the green-seeded cultivar BIS9805 and mapped a seed coat color locus to a 0.66 Mb interval on chr 4. Eight genes were located within this interval, of which *LOC106758748*, *LOC106758747*, and *LOC106759075* were differentially expressed between the two parents and were proposed as candidate genes. Thus, *LOC106758748* had previously been implicated in mung bean seed coat color, but the 0.66 Mb interval still contained multiple candidate genes and did not resolve which candidate was most strongly associated with the black-versus-green phenotype. Further improvement in mapping resolution and additional genetic and molecular evidence were therefore required to prioritize among these candidates.

Building on this previous work, reciprocal crosses were made between the black-seeded mung bean accession LZL115 and the green-seeded accession LZL156, and six F_2_ populations were developed to determine the inheritance of black seed coat color. Bulked segregant analysis sequencing (BSA-seq) was used to identify the genomic region associated with the target trait, after which KASP markers and expanded mapping populations were used to fine-map the locus. Parental sequence comparison, protein structure prediction, qRT-PCR analysis of developing seed coats, and haplotype analysis in a diverse mung bean panel were then integrated to prioritize the most likely candidate gene and evaluate candidate-region alleles associated with seed coat phenotype. This study therefore aimed to fine-map the locus associated with black seed coat color, prioritize the most likely candidate gene, and evaluate the potential utility of associated molecular markers for germplasm identification and marker-assisted selection.

## 2. Results

### 2.1. Genetic Analysis of the Black Seed Coat Trait

**Figure 1:**
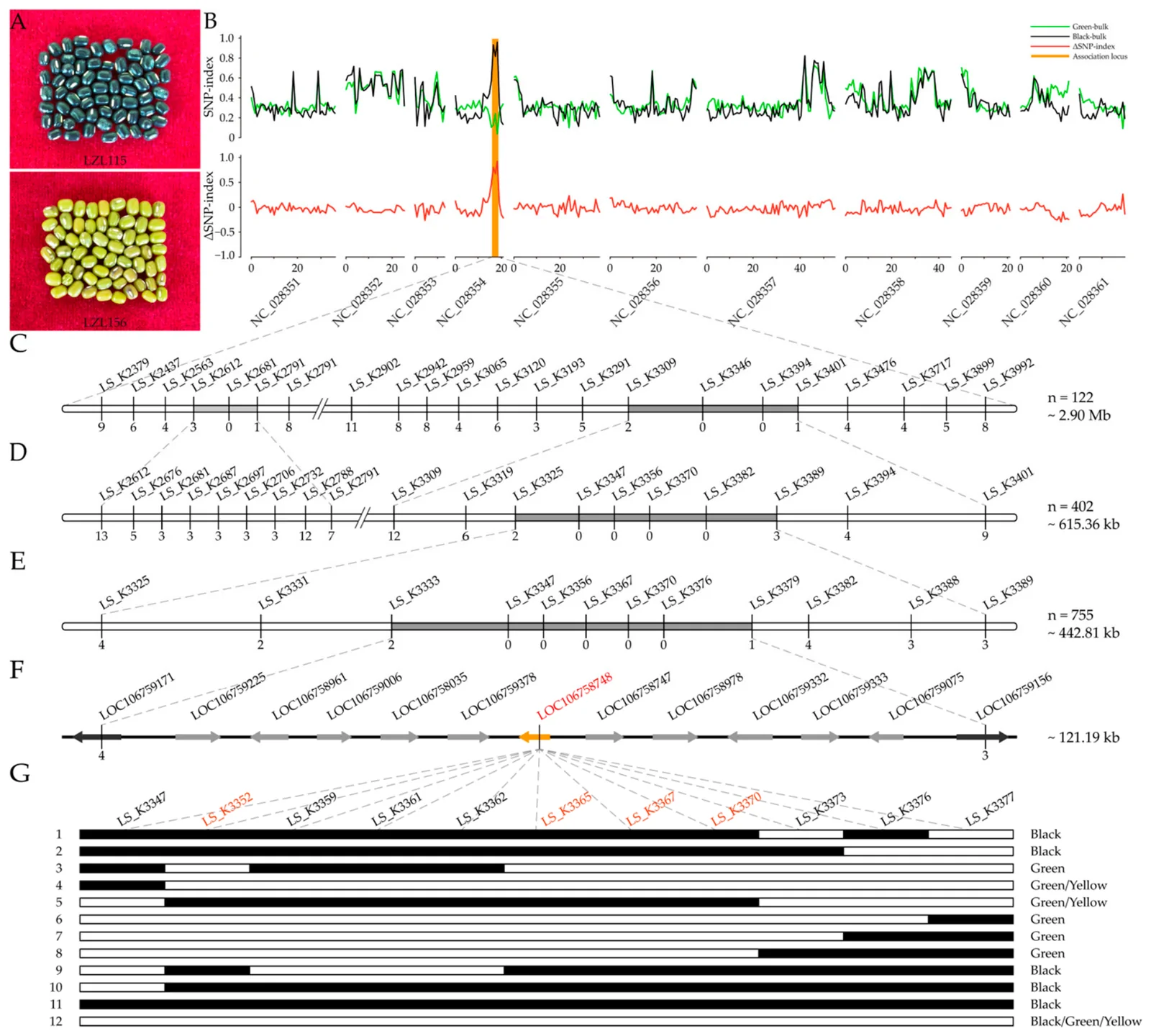
Phenotypic characterization, bulked segregant analysis sequencing, fine mapping, and haplotype analysis of the mung bean black seed coat gene *VrScL115*. (**A**) Seed coat phenotypes of the black-seeded parent LZL115 and the green-seeded parent LZL156. (**B**) Mapping of the seed coat color locus by BSA-seq. The *x*-axis indicates the physical positions of the 11 mung bean chromosomes, and the *y*-axis represents the SNP-index or ΔSNP-index values. (**C**) Initial mapping of the seed coat color locus using 122 F_2_ individuals. (**D**) Further mapping using 402 F_2_ individuals. (**E**) Fine mapping using 755 F_2_ individuals; the final 121.79 kb interval is delimited by LS_K3333 and LS_K3379. In (**C**–**E**), numbers below the markers indicate the numbers of recombinant individuals detected at the corresponding marker positions, and dashed lines indicate the corresponding regions between successive mapping stages. (**F**) Distribution of annotated genes within the approximately 121.79 kb candidate interval. Arrows indicate the predicted transcriptional directions of the genes. Arrows indicate the predicted transcriptional directions of the genes. (**G**) Haplotype analysis based on markers surrounding *LOC106758748*, showing the marker genotypes and corresponding seed coat color phenotypes of the identified haplotypes. Black rectangles correspond to homozygous or heterozygous genotypes of LZL115, whereas white rectangles represent homozygous genotypes of LZL156. Red labels highlight the candidate gene and co-segregating markers.

Phenotypic evaluation of mature seeds revealed a clear difference in seed coat color between the black-seeded parent LZL115 and the green-seeded parent LZL156 (Figure 1A). The six F_2_ populations derived from reciprocal crosses between the two parental lines comprised a total of 755 plants and showed clear segregation for seed coat color. These populations were derived from independent F_1_ plants and were therefore treated as independent segregation populations. Based on the seed coat phenotypes of mature seeds harvested from individual F_2_ plants, 559 plants were classified as black-seeded and 196 as green-seeded (Table 1). The observed segregation conformed to the expected Mendelian ratio of 3:1 (*χ*^2^ = 0.37, *p* = 0.54). Similarly, the segregation ratios in each of the six F_2_ populations were consistent with a 3:1 ratio, with *χ*^2^ values ranging from 0.39 to 0.89 and *p* values ranging from 0.35 to 0.53. No significant heterogeneity in the segregation pattern was detected among the six F_2_ populations (*χ*^2^ = 3.08, *p* = 0.69), supporting the pooled analysis of the 755 F_2_ plants (Table 1). These results indicated that, in the LZL115 × LZL156 genetic background, black seed coat color was dominant over green and was consistent with segregation of a single dominant locus, designated *VrScL115*.

### 2.2. Whole-Genome Resequencing and Variant Detection

Whole-genome resequencing was performed for the green-seeded parent LZL156, the black-seeded parent LZL115, the Green-bulk, and the Black-bulk using the Illumina sequencing platform. After quality filtering, a total of 9.80, 10.87, 14.99, and 14.97 Gb of clean bases were generated for LZL156, LZL115, the Green-bulk, and the Black-bulk, respectively, corresponding to 65,597,170, 72,789,188, 100,559,978, and 100,486,078 clean reads retained after filtering (Table 2). After alignment to the mung bean reference genome VC1973A v6.0, the mapping rates ranged from 97.88% to 99.67%. The mean sequencing depths were 20× and 22× for LZL156 and LZL115, respectively, and 31× for both segregant bulks. The proportions of the reference genome covered by at least five reads ranged from 94.36% to 96.75%. The Q30 values were 98.42%, 98.25%, 98.64%, and 98.45% for LZL156, LZL115, the Green-bulk, and the Black-bulk, respectively, while the corresponding GC contents were 34.30%, 34.90%, 32.96%, and 33.54%. Together, these quality-control metrics indicated high sequencing accuracy, stable base composition, high mapping efficiency, and sufficient genome coverage for the subsequent BSA-seq analysis.

After variant calling and quality filtering, 761,802 SNPs and 99,442 InDels, representing a combined total of 861,244 high-confidence variants, were retained for subsequent analysis (Table S1). SNPs and InDels were treated jointly in the subsequent BSA-seq analyses unless otherwise specified.

### 2.3. BSA-Seq Analysis and Preliminary Mapping of VrScL115

Both SNPs and InDels were included in the calculation of the SNP-index and ΔSNP-index. The BSA-seq analysis was based on a single comparison between the Black-bulk and Green-bulk constructed from the LZL156 × LZL115-A F_2_ population. Genome-wide analysis of the SNP-index profiles revealed a pronounced difference in allele frequency between the Black-bulk and Green-bulk on chr 4 (Figure 1B). The strongest association signal was located between 15,587,726 and 18,491,044 bp, defining a physical interval of approximately 2.90 Mb. Within this region, the SNP-index of the Black-bulk remained consistently high, with a mean value of approximately 0.90, whereas that of the Green-bulk was approximately 0.05. Correspondingly, the ΔSNP-index approached or exceeded 0.85 across the peak region and exceeded the simulation-derived 99% confidence interval generated by QTL-seq under the null hypothesis of no linkage. For downstream marker development, an absolute ΔSNP-index cutoff of 0.75 was used as a conservative operational criterion to prioritize variants within the associated region; this value was not treated as a second statistical significance threshold. The contrasting SNP-index patterns indicated that alleles derived from the black-seeded parent LZL115 were strongly enriched in the Black-bulk but were largely absent from the Green-bulk. Based on the continuous association signal and the marked difference in allele frequencies between the two bulks, the 15,587,726–18,491,044 bp region on chr 4 was preliminarily identified as the candidate interval containing *VrScL115*. This interval was subsequently used for KASP marker development and fine mapping of the locus underlying black seed coat color using expanded F_2_ populations.

### 2.4. KASP Marker Validation and Fine Mapping of VrScL115

Based on the BSA-seq results and the distribution of sequence variants within the candidate region, 22 polymorphic loci between 15,587,726 and 18,491,044 bp on chr 4 were selected for KASP marker development (Figure 1C, Table S2). These markers were initially used to genotype 122 F_2_ plants from the LZL156 × LZL115-A population. Three markers, LS_K2681, LS_K3346, and LS_K3394, cosegregated with the seed coat color phenotype. Recombination analysis provisionally defined two candidate intervals. The first was a 41.99 kb interval between LS_K2612 and LS_K2791, extending from 15,649,122 to 15,691,108 bp and containing LS_K2681. The second was a 615.36 kb interval between LS_K3309 and LS_K3401, extending from 17,318,219 to 17,933,574 bp and containing LS_K3346 and LS_K3394. These two intervals were separated by approximately 1.63 Mb.

To further resolve the target region, 12 additional KASP markers were developed within and around the two provisional intervals (Table S2). These markers were used to genotype 402 plants from three F_2_ populations derived from crosses between LZL156 and LZL115. Recombinant analysis excluded the first provisional interval and localized *VrScL115* to a 442.81 kb region between LS_K3325 and LS_K3389, corresponding to 17,351,156–17,793,969 bp on chr 4. This region was contained within the previously identified 17.32–17.93 Mb interval. Four markers, LS_K3346, LS_K3356, LS_K3367, and LS_K3382, cosegregated with the seed coat color phenotype in the 402 F_2_ plants (Figure 1D).

The mapping population was subsequently expanded to 755 plants from all six F_2_ populations, and six additional KASP markers were developed within the 442.81 kb interval (Table S2). Analysis of informative recombinants delimited *VrScL115* to a 121.79 kb interval between LS_K3333 and LS_K3379 (Table S3). Five markers, LS_K3346, LS_K3356, LS_K3367, LS_K3373, and LS_K3376, cosegregated completely with the seed coat color phenotype, and no recombination between these markers and the target locus was detected among the 755 F_2_ plants (Figure 1E).

### 2.5. Candidate Gene Identification and Sequence Analysis

According to the VC1973A v6.0 reference genome annotation, 11 genes were located within the 121.79 kb fine-mapped interval of *VrScL115* (Figure 1F). Comparative sequence analysis between LZL115 and LZL156 revealed no obvious polymorphisms in the coding regions or examined 5′ upstream regions of the other 10 genes, whereas *LOC106758748* contained multiple high-confidence sequence variants that consistently distinguished the two parental lines. On the basis of these sequence differences, together with its functional annotation, *LOC106758748* was prioritized for further analysis as the leading candidate gene for *VrScL115*. A total of 20 sequence variants were detected within its coding region, whereas no stable coding-sequence polymorphisms were identified in the other 10 annotated genes. The absence of coding-sequence polymorphisms in these 10 genes does not exclude possible regulatory, structural, intronic, untranslated-region, expression-level, or other functional variation affecting their activity. *LOC106758748* was also among the candidate genes previously proposed for mung bean seed coat color by Wang et al. [23]. Several of its coding-region variants were predicted to alter the encoded amino acid sequence, providing additional support for this prioritization.

To verify the sequence differences between the parental lines, the genomic sequence of *LOC106758748* was independently amplified from five individual plants of LZL115 and five individual plants of LZL156, and the LZL115- and LZL156-type alleles were independently confirmed by bidirectional Sanger sequencing (Table S3). The candidate variants were further validated by Sanger sequencing in 20 individual F_2_ plants, including 10 black-seeded and 10 green-seeded plants. All 10 green-seeded F_2_ plants were homozygous for the LZL156-type allele at the examined polymorphic sites. Among the 10 black-seeded F_2_ plants, three were homozygous for the LZL115-type allele and seven were heterozygous, carrying both the LZL115- and LZL156-type alleles. These results were consistent with the dominant inheritance of black seed coat color observed in the segregation analysis and independently confirmed the parental sequence differences and the candidate variants in individual F_2_ plants (Table S3).

**Figure 2:**
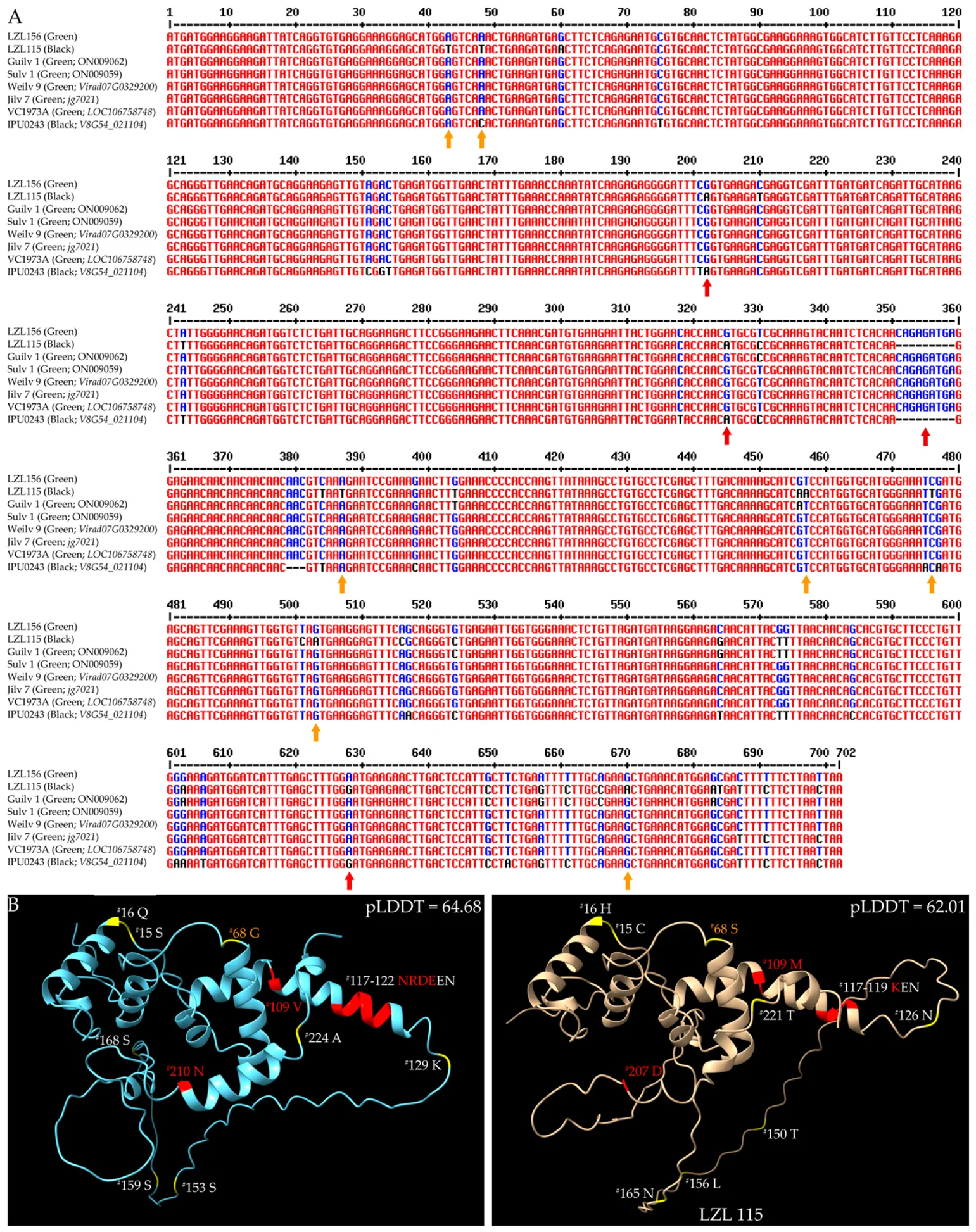
Sequence alignment and predicted protein structures of the candidate gene *LOC106758748.* (**A**) Alignment of *LOC106758748* coding sequences from seven mung bean (*Vigna radiata*) accessions and the black gram (*Vigna mungo*) accession IPU0243. Information in parentheses indicates seed coat color and the corresponding gene ID or NCBI accession number. Arrows indicate the 11 identified variation sites, with red arrows highlighting the variation sites corresponding to co-segregating markers. (**B**) Predicted protein structures of *LOC106758748* in LZL156 and LZL115. The “#” followed by a number indicates the corresponding amino acid position, and single-letter codes represent amino acid residues. Colored residue labels highlight amino acid differences corresponding to the sequence variations shown in (**A**). The sequence differences shown in (**A**) were derived from experimentally validated parental sequences, whereas the three-dimensional structures in (**B**) were computationally predicted using AlphaFold 3. The global pLDDT scores were 62.01 for LZL156 and 64.68 for LZL115.

Based on the annotated exon–intron structure of *LOC106758748* in the reference genome, the coding sequences of the two parental alleles were reconstructed from the corresponding genomic sequences. The reconstructed coding sequences were then aligned with the reference coding sequence and homologous sequences retrieved from the NCBI database for mung bean accessions and the black gram (*Vigna mungo*) accession IPU0243, which has a known black seed coat phenotype and contains a highly similar homolog of *LOC106758748* (Figure 2A). Among the 20 coding-region variants, 11 informative loci were selected based on parental polymorphism, predicted effects on the encoded amino acid sequence, allelic variation among the examined materials, and suitability of the flanking sequences for marker design. KASP markers were developed for these loci and designated LS_K3347, LS_K3352, LS_K3359, LS_K3361, LS_K3362, LS_K3365, LS_K3367, LS_K3370, LS_K3373, LS_K3376, and LS_K3377. These markers were subsequently used for haplotype analysis and evaluation of their association with seed coat color and potential utility for identifying the LZL115-associated haplotype (Figure 2A; Table S2).

### 2.6. Haplotype Analysis and Evaluation of Markers Associated with Black Seed Coat Color

The 11 KASP markers developed within the candidate region were used to genotype 246 mung bean accessions with well-defined seed coat color phenotypes (Table S4). The panel comprised 27 black-seeded, 210 green-seeded, and 9 yellow-seeded accessions, with no other seed coat color classes represented. Based on the multilocus genotype combinations, the accessions were classified into 12 haplotypes, which showed distinct distributions among the different seed coat color classes (Figure 1G).

At LS_K3352, LS_K3365, LS_K3367, and LS_K3370, 17 accessions were homozygous for the LZL115-type allele combination and four were heterozygous, and all 21 accessions exhibited black seed coats (Table S4). The LZL156-type combination was detected in 225 accessions, comprising 6 black-, 210 green-, and 9 yellow-seeded accessions. Thus, the LZL115-associated four-marker combination was present in 21 of 27 black-seeded accessions (77.8%) and absent from all 219 non-black accessions. When the presence of this marker combination was used to predict the black seed coat phenotype, sensitivity, specificity, positive predictive value, and negative predictive value were 77.8%, 100%, 100%, and 97.3%, respectively. A two-sided Fisher’s exact test confirmed a significant association between the four-marker combination and black seed coat color (*p* = 2.25 × 10^−25^), with a large association effect (Cramér’s V = 0.870).

The six black-seeded accessions lacking the LZL115-associated four-marker combination represented 22.2% of all black-seeded accessions. These exceptions may reflect alternative functional alleles at *VrScL115* or genetically distinct mechanisms controlling black seed coat color; although recombination between the tested markers and the functional variant cannot be excluded, the present data do not provide direct evidence for such recombination. Overall, the four-marker combination was strongly associated with black seed coat color in the present germplasm panel and may be useful for identifying accessions carrying the LZL115-associated haplotype.

### 2.7. Comparison of Deduced Amino Acid Sequences and Predicted Protein Structures

The coding sequences of *LOC106758748* in LZL115 and LZL156 were reconstructed from the corresponding genomic sequences according to the annotated exon–intron structure, and the deduced amino acid sequences were subsequently compared. Several amino acid differences were identified between the two parental proteins (Figure 2B). At position 109, LZL156 contained valine, whereas LZL115 contained methionine, resulting in a V109M substitution. A more pronounced sequence difference was observed in the region corresponding to residues 117–122 of LZL156. This region contained the six-amino-acid sequence NRDEEN in LZL156 but was represented by KEN in LZL115, resulting in three amino acid substitutions together with a net deletion of three residues. Because of this deletion, the downstream amino acid positions in LZL115 were shifted by three residues relative to those in LZL156. At the homologous site corresponding to residue 210 in LZL156 and residue 207 in LZL115, asparagine in LZL156 was replaced by aspartic acid in LZL115. This difference was designated N210D based on the LZL156 sequence numbering. These amino acid differences were derived from the experimentally validated sequence differences, whereas the following three-dimensional structural comparison was entirely computational.

The predicted three-dimensional structures of the LOC106758748-encoded proteins from LZL115 and LZL156 were generated using AlphaFold 3 and compared using UCSF ChimeraX v1.11.1 (Figure 2B). The global pLDDT scores were 64.68 for LZL115 and 62.01 for LZL156, indicating relatively low overall confidence in the predicted structures and requiring cautious interpretation of local structural differences. The two proteins showed a highly similar overall structural framework in the well-aligned core region. After iterative pruning, the root-mean-square deviation (RMSD) calculated from 111 matched atom pairs was 0.485 angstroms (Å), indicating close agreement within the well-aligned predicted core. In contrast, the RMSD across all 230 matched atom pairs was 15.729 Å, reflecting poor superposition of the predicted variable regions. Thus, the computational models suggest that the observed amino acid substitutions and deletion may affect the conformation of specific variable regions and potentially influence protein function, although this possibility requires experimental validation.

### 2.8. Candidate Gene Expression Analysis

**Figure 3:**
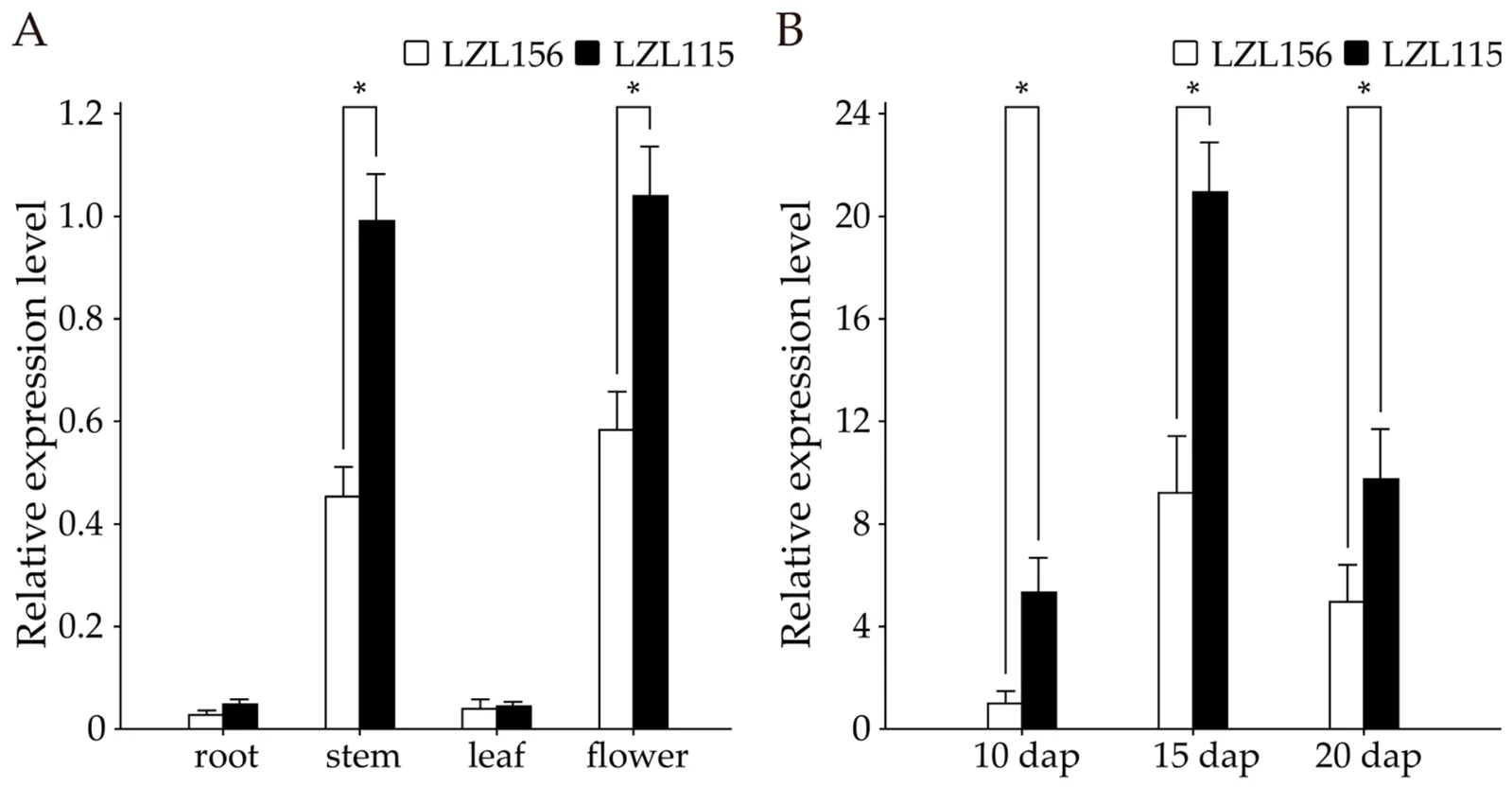
Expression patterns of the candidate gene *LOC106758748* in LZL156 and LZL115. (**A**) Relative expression levels of *LOC106758748* in the roots, stems, leaves, and flowers of the two parental lines. (**B**) Relative expression levels of *LOC106758748* in developing seed coats collected at 10, 15, and 20 days after pollination (dap). The qRT-PCR data were normalized to the expression of the reference gene *VrACTIN-3*. Values are presented as the mean ± standard error of three independent biological replicates. An asterisk (*) indicates a significant difference between LZL156 and LZL115 within the same tissue or developmental stage, as evaluated using a two-tailed Student’s *t*-test (*p* < 0.05).

The tissue-specific expression pattern of *LOC106758748* was examined in the roots, stems, leaves, and flowers of LZL156 and LZL115. *LOC106758748* showed relatively high expression in stems and flowers but low expression in roots and leaves. Its transcript levels in the stems and flowers of the black-seeded parent LZL115 were significantly higher than those in the green-seeded parent LZL156, whereas no significant differences between the two parental lines were detected in roots or leaves (Figure 3A).

To further investigate the relationship between *LOC106758748* expression and seed coat development, its transcript levels were examined in developing seed coats collected at 10, 15, and 20 dap. *LOC106758748* was expressed in the seed coats of both parental lines but showed consistently higher transcript levels in LZL115 than in LZL156 at all three developmental stages. In both parental lines, its expression increased from 10 to 15 dap and subsequently decreased at 20 dap. The highest transcript abundance was observed at 15 dap, when expression in LZL115 was significantly higher than that in LZL156. Although expression declined at 20 dap, it remained significantly higher in LZL115 than in LZL156 (Figure 3B).

These results demonstrated that *LOC106758748* exhibited tissue- and developmental stage-dependent expression. Its consistently higher expression in the developing seed coats of the black-seeded parent, together with the coding-sequence variation and haplotype association results, suggests that *LOC106758748* may be involved in black seed coat development and provides additional support for its prioritization as the leading candidate gene for *VrScL115*.

## 3. Discussion

Seed coat color is an important component of seed appearance in mung bean and directly influences commercial quality, consumer preference, and the breeding of market-oriented cultivars [2,23,24]. It is also associated with variation in bioactive compounds, particularly flavonoids and anthocyanins, and therefore has potential implications for the nutritional and functional value of mung bean seeds [5,25]. Previous studies have shown that the inheritance of black seed coat color varies among crosses, ranging from single-gene dominant or codominant inheritance to more complex genetic control, suggesting that the underlying genetic basis may depend on the parental combination and genetic background [15,26,27]. In the present study, six independent F_2_ populations were developed from reciprocal crosses between the black-seeded accession LZL115 and the green-seeded accession LZL156. In all six populations, the segregation of black- and green-seeded plants conformed to the expected 3:1 ratio, indicating that the phenotypic difference between the two parents was consistent with segregation of a single dominant locus specifically in the LZL115 × LZL156 genetic background. Similar segregation patterns were observed in the reciprocal crosses, and no obvious reciprocal-cross effect was detected. The locus controlling black seed coat color in this genetic background was therefore designated *VrScL115*. However, six of the 27 black-seeded accessions in the broader germplasm panel lacked the LZL115-associated four-marker haplotype, indicating that black seed coat color is not genetically uniform across mung bean and may involve alternative alleles or genetically distinct mechanisms in different backgrounds.

BSA-seq enables rapid genome-wide identification of trait-associated regions through comparison of allele-frequency differences between bulked DNA samples with contrasting phenotypes and is particularly suitable for qualitative traits controlled by major-effect loci [28,29,30]. In this study, BSA-seq initially mapped *VrScL115* to an approximately 2.90 Mb region on chr 4, and subsequent KASP marker development and recombinant screening progressively narrowed the interval to 121.79 kb. The major advance of this mapping strategy is therefore the substantial improvement in resolution, which reduced a broad association region to a compact interval containing only 11 annotated genes and enabled more focused candidate-gene evaluation. The five completely cosegregating markers represent tightly linked markers within the fine-mapped interval rather than causal variants, whereas potential functional variants were evaluated separately during subsequent candidate-gene analysis. Compared with the previously reported 0.66 Mb chr 4 interval associated with black-versus-green seed coat color [23], the 121.79 kb interval substantially narrows the positional evidence and provides a more precise framework for evaluating candidate genes. The first round of KASP-based linkage analysis using a relatively small mapping population provisionally identified two physically separated intervals. With increased marker density and expansion of the mapping population, the alternative interval was excluded and *VrScL115* was ultimately delimited to a single region. These results demonstrate that combining the rapid genome-wide screening capacity of BSA-seq with high-resolution linkage analysis based on expanded populations and informative recombinants can effectively improve mapping accuracy and resolution. A technical limitation of the present study is the relatively modest sequencing depth used for BSA-seq. The parental lines were sequenced at approximately 20–22× depth and the two segregant bulks at approximately 31× depth. Because allele-frequency estimates in pooled sequencing are dependent on read coverage, moderate sequencing depth may increase sampling variation and reduce the precision of SNP-index estimates, particularly in genomic regions with uneven or relatively low coverage [29,30]. Therefore, although the sequencing data were sufficient to detect the major association signal, the precision of allele-frequency estimates should be interpreted with appropriate caution.

Previous genetic and genomic studies have identified mung bean seed coat color-associated loci on chr 2, 3, 4, 5, and 7, indicating that the genetic architecture of this trait varies among populations and genetic backgrounds [11,21,23]. Several candidate genes have been proposed, including *VrMYB113*, *LOC106758748*, *LOC106758747*, and *LOC106759075* on chr 4 and *Vrsf3′h1* on chr 5 [11,23]. In particular, Wang et al. [23] mapped a black-versus-green seed coat color locus to a 0.66 Mb interval on chr 4 and proposed *LOC106758748*, *LOC106758747*, and *LOC106759075* as candidate genes. The present study further reduced the target interval to 121.79 kb and found that *LOC106758748* was the only annotated gene in this interval containing high-confidence coding-sequence polymorphisms between LZL115 and LZL156, thereby providing stronger positional and sequence-based support for its prioritization. Nevertheless, the absence of coding polymorphisms does not by itself functionally exclude other genes, because regulatory, structural, intronic, untranslated-region, or expression-level variation could contribute to the phenotype without altering coding sequence. Accordingly, the present data prioritize *LOC106758748* rather than definitively ruling out all alternative candidates.

Sequence variation and differential gene expression can provide complementary evidence for identifying genes associated with seed coat pigmentation [23,31,32]. In the present study, the genomic alleles of *LOC106758748* were sequenced in LZL115 and LZL156, and multiple coding-region polymorphisms were identified between the two parental lines. Relative to LZL156, the deduced LOC106758748 protein of LZL115 contained the V109M and N210D substitutions, together with replacement of the NRDEEN segment at residues 117–122 by KEN, resulting in a net deletion of three amino acids. These sequence differences were additionally validated in individual F_2_ plants by Sanger sequencing. Comparison with homologous sequences from materials with known seed coat phenotypes provided further comparative evidence, although the *Vigna mungo* accession IPU0243 should be regarded only as an interspecific reference rather than direct evidence for a causal variant in *V. radiata*.

The AlphaFold-derived structural models indicated a conserved core but predicted differences in some variable regions between the LZL115 and LZL156 proteins. These predicted structural differences are computational observations and do not demonstrate a functional consequence of the amino acid substitutions or deletion. Expression analysis showed that *LOC106758748* was consistently more highly expressed in developing seed coats of LZL115 than of LZL156 at 10, 15, and 20 days after pollination. This expression difference suggests a possible role in seed coat pigmentation, but differential expression between the two parental genotypes is correlative and could represent either a cause or a consequence of phenotypic divergence. Taken together, fine mapping, coding-sequence polymorphisms, individual-plant Sanger validation, predicted structural differences, expression differences, and haplotype association provide convergent support for *LOC106758748* as the leading candidate gene for *VrScL115*, but none of these analyses constitute direct functional proof; in vivo functional validation remains necessary to establish causality. Potential regulatory variation outside coding regions also cannot be excluded. Variants in promoters, introns, untranslated regions, or other cis-regulatory regions may influence gene expression without altering the encoded protein, and non-coding RNAs may also contribute to seed coat pigmentation [33,34,35].

Molecular markers tightly linked to major loci can facilitate germplasm screening and marker-assisted selection by enabling the rapid identification of favorable alleles. Previous genetic studies have identified several loci and sequence variants associated with seed coat color in mung bean, providing a basis for the development of trait-associated molecular markers [11,21,23]. However, marker performance may vary among genetic backgrounds and therefore requires evaluation in diverse germplasm. In the present study, the LZL115-associated allele combination at LS_K3352, LS_K3365, LS_K3367, and LS_K3370 was detected in 21 of 27 black-seeded accessions (77.8%) but in none of the 219 non-black accessions, corresponding to a sensitivity of 77.8%, specificity of 100%, PPV of 100%, and NPV of 97.3%. The six black-seeded accessions lacking this marker combination may carry alternative functional alleles at *VrScL115* or genetically distinct loci or mechanisms controlling black seed coat pigmentation. Therefore, the four-marker combination should be regarded as a set of markers associated with the LZL115-type black seed coat haplotype rather than as a universally diagnostic marker set, and further validation in independent germplasm populations is required before broader application in marker-assisted breeding.

LOC106758748 is annotated as encoding the R2R3-MYB transcription factor *VrMYB90*, making it a biologically plausible candidate for involvement in mung bean seed coat pigmentation [23,31,36,37]. R2R3-MYB transcription factors are major regulators of flavonoid and anthocyanin biosynthesis and often function with bHLH and WD40 proteins in MYB–bHLH–WD40 regulatory complexes [37,38,39,40]. Previous studies have reported that *VrMYB90* can promote anthocyanin biosynthesis by activating *VrDFR* and *VrANS*, interacting with *VrbHLHA*, and influencing *VrANR* expression and metabolic flux between anthocyanin and proanthocyanidin biosynthesis [31,36,41]. The new evidence generated in the present study consists of higher-resolution mapping, parental coding-sequence variation, individual-plant Sanger validation, predicted structural differences, expression differences in developing seed coats, and haplotype association. Previous studies detected *VrMYB90*-associated pigmentation in the hypocotyl, stem, petiole, and flower, whereas the present study detected clear expression differences in isolated developing seed coats. However, we did not directly test transcriptional activation, DNA binding, or protein–protein interactions in developing mung bean seed coats; therefore, whether *LOC106758748* functions through a canonical MYB–bHLH–WD40 complex in this tissue remains a hypothesis requiring direct experimental validation.

In conclusion, the dominant locus *VrScL115* controlling black seed coat color was fine-mapped to a 121.79 kb interval on chr 4. *LOC106758748*, encoding the R2R3-MYB transcription factor VrMYB90, was prioritized as the leading candidate gene based on convergent evidence from sequence variation, expression analysis, protein structure prediction, and haplotype association. However, these findings provide strong genetic and molecular support rather than direct functional proof, and further in vivo functional validation is required to establish the causal role of LOC106758748 in black seed coat formation. The marker combination LS_K3352, LS_K3365, LS_K3367, and LS_K3370 showed strong association with black seed coat color and has potential utility for germplasm identification and marker-assisted selection.

## 4. Materials and Methods

### 4.1. Plant Materials

The black-seeded mung bean accession LZL115 and the green-seeded accession LZL156 were used as parental lines. Reciprocal crosses, LZL115 × LZL156 and LZL156 × LZL115, were performed to produce F_1_ plants. Independent F_1_ plants were self-pollinated to generate six F_2_ populations comprising a total of 755 plants (Table 1). These populations were used for genetic analysis, BSA-seq, and fine mapping with Kompetitive Allele-Specific PCR (KASP) markers.

In addition, a panel of 246 mung bean accessions was used for seed coat color evaluation, haplotype analysis, and evaluation of markers associated with black seed coat color (Table S4). All plant materials were provided by the Liaoning Provincial Germplasm Resource Bank and grown at the experimental station in Shenyang, Liaoning Province, China (41.82° N, 123.54° E), during the 2023 and 2024 growing seasons. Field management, including land preparation, sowing, fertilization, irrigation, weed control, and pest and disease management, followed the Liaoning provincial standard Technical Regulations for Mung Bean Seed Production [42].

### 4.2. Phenotypic Evaluation and Genetic Analysis of Seed Coat Color

Seed coat color was evaluated using fully mature and naturally dried seeds. Seeds from the parental lines and germplasm accessions were harvested separately by accession. For each F_2_ plant, mature self-pollinated seeds were harvested separately, and the seed coat phenotype of the corresponding F_2_ plant was determined from the seeds produced by that plant.

All available mature seeds harvested from each F_2_ plant were visually examined under uniform natural light conditions rather than using a fixed number of seeds per plant. Because the seed coat is maternally derived, seeds harvested from the same F_2_ plant showed a consistent seed coat phenotype and were used to assign the phenotype of that individual plant. The primary seed coat phenotypes were classified as black or green, whereas accessions showing other colors or pigmentation patterns were recorded separately. Samples with unclear or inconsistent appearance were re-examined under the same conditions, and only plants with an unambiguous seed coat phenotype were used for bulk construction and recombinant screening [23].

The observed numbers of black- and green-seeded F_2_ plants were compared with the expected Mendelian segregation ratio using a chi-square goodness-of-fit test. The inheritance pattern of black versus green seed coat color was inferred from the segregation results. Phenotypic records for the 246 accessions were subsequently used for haplotype analysis and evaluation of markers associated with black seed coat color.

### 4.3. Genomic DNA Extraction and Bulk Construction

Young leaves were collected from LZL115, LZL156, individual F_2_ plants, and the 246 mung bean accessions. Genomic DNA was extracted using the E.Z.N.A.^®^ HP Plant DNA Kit (D2485-03; Omega Bio-tek, Norcross, GA, USA) according to the manufacturer’s instructions. DNA integrity was examined by 1.2% agarose gel electrophoresis, and DNA purity and concentration were evaluated using a NanoDrop 2000/2000c spectrophotometer (Thermo Fisher Scientific, Waltham, MA, USA). DNA samples used for whole-genome resequencing had a concentration of at least 50 ng/µL, whereas DNA used for KASP genotyping and PCR assays was normalized to approximately 10 ng/µL. DNA purity was assessed using the NanoDrop A260/A280 ratio, and DNA integrity was assessed by 1.2% agarose gel electrophoresis.

Based on the seed coat phenotypes of seeds harvested from individual plants in the LZL156 × LZL115-A F_2_ population, 20 plants producing uniformly black-coated seeds and 20 plants producing uniformly green-coated seeds were selected for bulk construction. DNA samples were normalized to an equal concentration and mixed in equal volumes to construct a black-seeded bulk (Black-bulk) and a green-seeded bulk (Green-bulk), ensuring an equal DNA contribution from each selected plant. BSA-seq was performed only for this pair of contrasting bulks from the LZL156 × LZL115-A population and was used as the initial genome-wide locus-discovery step. The remaining F_2_ populations were not subjected to independent BSA-seq analyses; instead, they were subsequently incorporated into KASP-based linkage analysis and recombinant screening to validate and progressively refine the candidate interval. Thus, the initial BSA-seq signal was derived from one F_2_ population, whereas the final fine-mapping result was supported by the expanded set of six F_2_ populations.

### 4.4. Read Processing, Sequence Alignment, and Variant Detection

Raw sequencing reads were quality-filtered using fastp v0.23.4 [43]. Quality filtering used the following parameters: --qualified_quality_phred 15, --unqualified_percent_limit 40, --n_base_limit 5, and --length_required 15; the low-complexity filter was not enabled. Adapter-containing reads, reads with more than 40% bases below Q15, reads containing more than five undetermined bases, and reads shorter than 15 bp after filtering were removed.

Clean paired-end reads were aligned to the mung bean reference genome VC1973A v6.0 using bwa-mem2 v2.2.1 [44] with the mem algorithm. The principal alignment parameters were the BWA-MEM defaults used by bwa-mem2: minimum seed length = 19 (-k 19), band width = 100 (-w 100), Z-dropoff = 100 (-d 100), reseeding ratio = 1.5 (-r 1.5), match score = 1 (-A 1), mismatch penalty = 4 (-B 4), gap-open penalty = 6 (-O 6), gap-extension penalty = 1 (-E 1), clipping penalty = 5 (-L 5), and minimum output alignment score = 30 (-T 30). Alignment files were converted to BAM format, sorted, and indexed using SAMtools v1.18 [45]. PCR duplicates were identified using the MarkDuplicates module of the Genome Analysis Toolkit (GATK v4.6) [46]; duplicate reads were marked but not removed.

In this study, InDel variants were treated together with SNPs and included in subsequent analyses. Variant calling was conducted chromosome by chromosome using GATK HaplotypeCaller [46]. Chromosome-specific intervals were supplied with the -L option, and standard HaplotypeCaller settings were retained, including a minimum base quality score of 10 and a minimum phred-scaled calling confidence of 30. The raw variant dataset was filtered using VCFtools v0.1.17 [47] with --minDP 5, --max-missing 1.0, and --maf 0.01. Thus, variants were retained only when each sample had a read depth of at least 5×, no genotype was missing across the parental lines and bulks, and the minor allele frequency was at least 0.01. Only high-confidence variants showing reliable allele-depth information were used in the subsequent BSA-seq analysis.

### 4.5. BSA-Seq Analyses and Candidate Region Identification

For each high-confidence polymorphic locus, the SNP-index was calculated separately for the Black-bulk and Green-bulk. The SNP-index was defined as the proportion of reads carrying the allele derived from the black-seeded parent LZL115 among the total reads covering that locus [28,29]. The ΔSNP-index was calculated by subtracting the SNP-index of the Green-bulk from that of the Black-bulk. Positive ΔSNP-index values indicated enrichment of the LZL115-derived allele in the Black-bulk, whereas values close to zero indicated little difference in allele frequency between the two bulks.

Genome-wide distributions of the SNP-index and ΔSNP-index were calculated and initially visualized using the qtlplot module implemented in QTL-seq v2.2.3 [48]. The analysis specified an F_2_ population (filial generation = 2) with 20 individuals in each bulk. The qtlplot parameters were a 2000 kb sliding window, a 100 kb step size, minimum and maximum variant depths of 8 and 250, respectively, a minimum SNP-index of 0.3, and 5000 simulation replicates. Under the null hypothesis of no linkage, QTL-seq simulated the ΔSNP-index distribution using the bulk size, filial generation, and read-depth information and generated 95% and 99% confidence intervals. Regions in which the smoothed ΔSNP-index exceeded the simulation-derived 99% confidence interval were considered statistically supported association regions. Within these regions, variants with |ΔSNP-index| ≥ 0.75 were prioritized for marker development as an operational filtering criterion. The resulting figures were further edited and refined using Adobe Illustrator 2026 (Adobe Inc., San Jose, CA, USA).

Sequence variants within the candidate region were functionally annotated using SnpEff v5.2 [49]. A custom SnpEff database was constructed using the VC1973A v6.0 reference genome sequence and its corresponding gff/gtf annotation file. The genomic position, mutation type, predicted coding effect, and potential influence of each variant on the encoded protein were recorded.

### 4.6. KASP Marker Development and Fine Mapping

Polymorphic loci within the BSA-seq candidate region were selected for KASP marker development. Candidate loci were required to be homozygous and polymorphic between LZL115 and LZL156 and to show clear differences in allele frequency between the Black-bulk and Green-bulk. Marker spacing across the candidate region, sequence quality, uniqueness of the flanking sequence, and expected genotyping performance were also considered. Variants located in repetitive regions or having non-specific, low-quality, or ambiguous flanking sequences were excluded. Flanking sequences extending 300 bp upstream and downstream of each candidate variant were extracted using TBtools-II v2.027 [50]. Sequence uniqueness was evaluated by local BLAST searches implemented in TBtools-II v2.027 against the reference genome. KASP primers for the filtered candidate loci were then designed using the KASP Primers Genotyping Assay Design online platform (https://primerdigital.com/tools/kasp.html (accessed on 25 December 2025)). Each assay contained two allele-specific forward primers and one common reverse primer. The two allele-specific primers carried the standard FAM (5′-GAAGGTGACCAAGTTCATGCT-3′) and HEX (5′-GAAGGTCGGAGTCAACGGATT-3′) tails at their 5′ ends, respectively (Table S2).

KASP genotyping was performed using the Douglas Scientific Array Tape platform. Each reaction had a total volume of approximately 1.6 µL and contained 0.8 µL genomic DNA, 0.022 µL primer assay mixture, 0.4 µL KASP V4.0 2× Mastermix 1536 (LGC Biosearch Technologies, Shanghai, China), and 0.4 µL ddH_2_O. PCR amplification was conducted using a Soellex thermal cycler (Douglas Scientific, Alexandria, MN, USA) with the following program: initial denaturation at 94 °C for 15 min; 10 touchdown cycles of 95 °C for 20 s and 65 °C for 60 s, with the annealing temperature decreasing by 0.6 °C per cycle; followed by 26 cycles of 94 °C for 20 s and 55 °C for 60 s; and a final hold at 4 °C. Endpoint fluorescence was detected using an Araya fluorescence detection system (part of the Douglas Scientific Array Tape Platform). Genotypes were automatically classified from endpoint-fluorescence clusters and then visually inspected using the genotyping data analysis module of Kraken™ laboratory workflow management software v2.0.8 (Douglas Scientific/LGC Biosearch Technologies). The polymorphic KASP markers were first used to genotype the parental lines and F_2_ plants with unambiguous seed coat phenotypes. Recombinant individuals were identified by comparing marker genotypes with the seed coat phenotypes assigned to individual F_2_ plants. Additional markers were subsequently developed within the recombinant interval, and the target locus was progressively delimited according to the positions of informative recombination events. The integrated genetic and physical maps were prepared using Adobe Illustrator 2026.

### 4.7. Candidate Gene Annotation and Sequence Analysis

Based on the fine-mapping results, genes within the candidate interval were subjected to functional annotation and sequence variation analysis. Key candidate genes were selected by integrating gene annotation information, variant types between the parental lines, and their potential involvement in pigment biosynthesis, transport, or transcriptional regulation. The gene sequence of each candidate gene was determined according to the reference genome annotation file, and gene-specific primers were designed to amplify candidate gene sequences from the black-seeded and green-seeded parental lines (Table S5). For sequence validation, five individual plants of each parental line (LZL115 and LZL156), together with 10 black-seeded and 10 green-seeded F_2_ plants, were independently sampled, and the corresponding candidate regions were amplified and subjected to bidirectional Sanger sequencing using the same gene-specific primers.

All primers were synthesized by Sangon Biotech (Shanghai, China). PCR amplification was performed using a GeneAmp 9700 thermal cycler (Applied Biosystems, Foster City, CA, USA). The total reaction volume was 10 µL, containing 1 µL genomic DNA approximately 20 ng/µL, 0.4 µL forward primer, 0.4 µL reverse primer, 5 µL 2× Taq PCR Master Mix (Tiangen Biotech, Beijing, China), and 3.2 µL ddH_2_O. The PCR program was as follows: initial denaturation at 94 °C for 4 min; 40 cycles of denaturation at 94 °C for 30 s, annealing at an appropriate temperature for 30 s, and extension at 72 °C for 1 min; followed by a final extension at 72 °C for 10 min.

After purification, the PCR products were sent to Sangon Biotech (Shanghai, China) for Sanger sequencing. For the F_2_ validation, the resulting sequences were aligned with the corresponding sequences of LZL115 and LZL156 to determine the genotypes of the candidate variants in each individual plant. The sequencing results were first searched against the NCBI database using BLAST to identify homologous sequences, and the potential functions of candidate genes were preliminarily inferred based on homologous sequence annotations. Subsequently, the DNA sequences of candidate genes were extracted according to the reference genome annotation information, and the sequences from the black-seeded parent, green-seeded parent, and reference DNA were imported into MultAlin (http://multalin.toulouse.inra.fr/multalin/ (accessed on 15 March 2026)) for multiple sequence alignment to identify sequence differences between the parental lines at the cDNA level. In combination with CDS and protein-coding sequence information, candidate variants were further evaluated to determine whether they caused synonymous mutations, nonsynonymous mutations, frameshift mutations, premature termination, or other changes potentially affecting gene function. These variants were then used for subsequent haplotype analysis and candidate gene variation assessment. For candidate genes with variants potentially affecting the encoded protein sequence, protein structures were predicted using AlphaFold 3 (https://alphafoldserver.com/welcome (accessed on 20 March 2026)) and visualized using ChimeraX v1.11.1 [51]. These structural models were used only as supportive computational predictions for comparative visualization.

### 4.8. Marker Genotyping and Haplotype Analysisr

KASP markers tightly linked to seed coat color within the candidate interval were applied to genotype 246 mung bean accessions. For each accession, young leaves were collected from a representative individual plant for genomic DNA extraction. The consistency between marker genotypes and seed coat color phenotypes was then analyzed in combination with phenotypic records of mature seeds.

Haplotypes were classified based on the genotype combinations of KASP markers within the candidate interval in the natural accessions, and the associations between different haplotypes and seed coat color phenotypes were compared. Markers that showed high consistency with the black seed coat phenotype and high predictive accuracy in natural accessions were identified as associated molecular markers for the black seed coat trait in mung bean. The association between the LZL115-associated marker combination and black versus non-black seed coat phenotype was evaluated using a two-sided Fisher’s exact test, and the strength of association was quantified using Cramér’s V. Sensitivity, specificity, positive predictive value (PPV), and negative predictive value (NPV) were calculated to evaluate marker performance.

### 4.9. RNA Extraction and qRT-PCR Analysis

To compare expression of the candidate gene between materials with different seed coat colors, the black-seeded and green-seeded parental lines were selected for seed coat tissue sampling during seed development. At the flowering stage, root, stem, leaf, and flower tissues were separately collected from healthy plants of both parental lines to examine the tissue-specific expression pattern of the candidate gene. Seed coat tissues were collected from developing seeds at 10, 15, and 20 days after pollination (dap) [23]. During sampling, seed coats were rapidly dissected, and three biological replicates were collected at each time point, with sufficient seed coat tissue collected for each replicate. All samples were immediately frozen in liquid nitrogen and stored at −80 °C until further use.

Total RNA was extracted from all collected tissue samples using the FastPure Universal Plant Total RNA Isolation Kit (RC411, Vazyme, Nanjing, China), and first-strand cDNA was synthesized using the HiScript II 1st Strand cDNA Synthesis Kit (+gDNA wiper) (R212, Vazyme, Nanjing, China). All procedures were performed according to the manufacturer’s instructions. Gene-specific primers for qRT-PCR were designed based on the candidate gene sequences using NCBI Primer-BLAST (https://www.ncbi.nlm.nih.gov/tools/primer-blast/ (accessed on 22 March 2026)), with all primer sequences provided in Table S5. VrACTIN-3 was used as the internal reference gene because the same reference gene had been used for normalization in a previous mung bean study examining *VrMYB90* expression [23].

The qRT-PCR reactions were prepared using 2× TransStart^®^ Top Green qPCR SuperMix (TransGen Biotech, Beijing, China) and performed on an Applied Biosystems QuantStudio™ 5 Real-Time PCR Instrument. The amplification program was as follows: 50 °C for 2 min and 95 °C for 10 min, followed by 40 cycles of 95 °C for 5 s and 60 °C for 34 s. Three biological replicates were included for each sample, and each biological replicate contained three technical replicates. Technical replicates were averaged for each biological replicate, and the biological replicate was used as the statistical unit. The relative expression level of the candidate gene was calculated using the 2^−ΔΔCt^ method [52], and the results were visualized using Chiplot (https://www.chiplot.online/ (accessed on 1 July 2026)) and Adobe Illustrator 2026.

### 4.10. Statistical Analysis

Statistical analyses were performed using R software v4.5.2 (R Foundation for Statistical Computing, Vienna, Austria). The experimental unit was an individual F_2_ plant for segregation and fine-mapping analyses, an individual accession for the 246-accession haplotype analysis, and an independently collected biological replicate for qRT-PCR analysis. For each of the six F_2_ populations, the observed black:green segregation was tested against the expected 3:1 ratio using a chi-square goodness-of-fit test with 1 degree of freedom. A heterogeneity chi-square test was used to evaluate whether segregation differed among the six independent populations, and the pooled 755-plant analysis was used as an overall summary when no significant heterogeneity was detected.

For the 246-accession dataset, association between the LZL115-associated four-marker combination and black versus non-black seed coat phenotype was evaluated using a two-sided Fisher’s exact test. Association strength was quantified using Cramér’s V, and marker performance was summarized by sensitivity, specificity, PPV, and NPV. For qRT-PCR, the three technical replicates were averaged within each of the three biological replicates, and statistical comparisons were performed on biological-replicate values rather than technical replicates. Pairwise comparisons between LZL115 and LZL156 within the same tissue or developmental stage were conducted using a two-tailed independent-samples Student’s *t*-test, with *p* < 0.05 considered statistically significant.

## 5. Conclusions

This study fine-mapped the dominant locus VrScL115, associated with black seed coat color in the LZL115 × LZL156 genetic background. Based on BSA-seq, KASP marker development, and recombinant screening, *VrScL115* was narrowed from an initial 2.90 Mb region to a 121.79 kb interval on chromosome 4. Among the 11 annotated genes in this interval, *LOC106758748*, encoding the R2R3-MYB transcription factor *VrMYB90*, was prioritized as the leading candidate based on coding-sequence polymorphisms, predicted structural differences, and higher expression in developing seed coats of the black-seeded parent. These results provide substantial genetic and molecular support for *LOC106758748*, although direct in vivo functional validation is still required to establish causality. The LZL115-associated four-marker combination at LS_K3352, LS_K3365, LS_K3367, and LS_K3370 was strongly associated with black seed coat color and has potential utility for germplasm identification and marker-assisted selection. Further validation in independent germplasm populations is required before broader breeding application.

## Figures and Tables

**Table 1:** Genetic segregation of seed coat color in the six F_2_ populations derived from reciprocal crosses between LZL156 and LZL115.

Parent or Cross	Generation	Amount	Observed Number		Chi-Squared Tests ^1^		
			Black	Green	Expected Ratio	*χ* ^2^	*Ρ*
LZL156 × LZL115–A	F_2_	122	87	35	3:1	0.89	0.35
LZL115 × LZL156–B	F_2_	143	104	39	3:1	0.39	0.53
LZL156 × LZL115–C	F_2_	137	99	38	3:1	0.55	0.46
LZL115 × LZL156–D	F_2_	98	77	21	3:1	0.67	0.41
LZL115 × LZL156–E	F_2_	139	108	31	3:1	0.54	0.46
LZL156 × LZL115–F	F_2_	116	84	32	3:1	0.41	0.52
LZL115 × LZL156	F_2_	755	559	196	3:1	0.37	0.54

^1^ The expected segregation ratio was 3:1 for the dominant and recessive phenotypes. A *χ*^2^ test was used to evaluate goodness of fit, with *χ*^2^ < 3.84 (*p* > 0.05) indicating consistency with the expected ratio. No multiple-testing correction was applied because each population represented an independent test of the predefined 3:1 Mendelian segregation hypothesis.

**Table 2:** Summary of whole-genome resequencing data for the parental lines and segregant bulks.

Sample	Clean Bases (Gb)	Clean Reads	Q30 (%)	GC (%)	Mapping Rate (%)	Mean Depth (×)	Genome Coverage at ≥5× (%)
LZL156	9.80	65,597,170	98.42	34.30	98.48	20	94.36
LZL115	10.87	72,789,188	98.25	34.90	97.88	22	94.41
Green-bulk	14.99	100,559,978	98.64	32.96	99.67	31	96.75
Black-bulk	14.97	100,486,078	98.45	33.54	99.63	31	96.71

## Data Availability

The original contributions presented in this study are included in the article/Supplementary Material. Further inquiries can be directed to the corresponding author(s).

## Supplementary Materials

The following supporting information can be downloaded at: https://www.mdpi.com/article/10.3390/plants15172594/s1, Table S1: Distribution of SNPs and InDels across the 11 mung bean chromosomes in the parental lines and segregant bulks; Table S2: The KASP primers used in this study; Table S3: Flanking-marker genotypes, seed coat phenotypes, and Sanger sequencing validation of selected F_2_ individuals for fine mapping of VrScL115; Table S4: Detection of markers LS_K3352, LS_K3365, LS_K3367, and LS_K3370 in 246 mung bean accessions; Table S5: Primers used for LOC106758748 amplification and qRT-PCR analysis.
